# Fragility of ER homeostatic regulation underlies haploid instability in human somatic cells

**DOI:** 10.1016/j.jbc.2024.107909

**Published:** 2024-10-19

**Authors:** Sumire Ishida-Ishihara, Kan Yaguchi, Sena Miura, Ryoto Nomura, QiJiao Wang, Koya Yoshizawa, Kimino Sato, Guang Yang, Krisztina Veszelyi, Gabor Banhegyi, Eva Margittai, Ryota Uehara

**Affiliations:** 1 Graduate School of Life Science, Hokkaido University, Sapporo, Japan; 2 Faculty of Advanced Life Science, Hokkaido University, Sapporo, Japan; 3 Institute of Translational Medicine, Semmelweis University, Budapest, Hungary; 4 Institute of Biochemistry and Molecular Biology, Semmelweis University, Budapest, Hungary

**Keywords:** ploidy, cell death, ER stress, proteostasis, HAP1 cell

## Abstract

Mammalian somatic cells are generally unstable in the haploid state, resulting in haploid-to-diploid conversion within a short time frame. However, cellular and molecular principles that limit the sustainability of somatic haploidy remain unknown. In this study, we found the haploidy-linked vulnerability to endoplasmic reticulum (ER) stress as a critical cause of haploid intolerance in human somatic cells. Pharmacological induction of ER stress selectively induced apoptosis in haploid cells, facilitating the replacement of haploids by coexisting diploidized cells in a caspase-dependent manner. Biochemical analyses revealed that unfolded protein response (UPR) was activated with similar dynamics between haploids and diploids upon ER stress induction. However, haploids were less efficient in solving proteotoxic stress, resulting in a bias toward a proapoptotic mode of UPR signaling. Artificial replenishment of chaperone function substantially alleviated the haploidy-linked upregulation of proapoptotic signaling and improved haploid cell retention under tunicamycin-induced ER stress. These data demonstrate that the ER stress-driven haploid instability stems from inefficient proteostatic control that alters the functionality of UPR to cause apoptosis selectively in haploids. Interestingly, haploids suffered a higher level of protein aggregation even in unperturbed conditions, and the long-term stability of the haploid state was significantly improved by alleviating their natural proteotoxicity. Based on these results, we propose that the haploidy-specific vulnerability to ER stress creates a fundamental cause of haploid intolerance in mammalian somatic cells. Our findings provide new insight into the principle that places a stringent restriction on the evolution of animal life cycles.

Ploidy, the genome copy number within a cell, is a biological trait that critically influences life functions at cellular and organismal levels. Life cycles in plants and fungi accommodate diversity in ploidy states. In contrast, animals, especially most mammals, invariably have a diplontic life cycle, where the multicellular somatic stage is restricted to the diploid state (*i.e.*, each cell possesses two genome copies). Mammalian haploid somatic cells occasionally arising from irregular biological events, such as parthenogenesis or tumorigenesis, cause developmental defects or tissue homeostatic disruption, respectively (1). This intolerance to haploidy imposes strict limits on the flexibility of mammalian life cycle evolution.

Mammalian haploid somatic cells are generally unstable and prone to cell division failure through frequent centrosome loss, which converts them to diploid through whole-genome duplication (1, 2, 3, 4, 5). Besides mitotic instability, haploid cells suffer poorer proliferative potential than their diploidized derivatives (5, 6). As a result, the haploid cell population is eventually replaced by a diploid population during consecutive cell proliferation, either *in vitro* or *in vivo* (5, 7). Having a single allele of all genes, haploid cells have greatly contributed to the development of molecular genetics and bioengineering, especially in microorganisms (8). However, the haploidy-linked problems described above limit the utilities of haploid cells in mammals. These multifaceted defects in haploid cells indicate that various aspects of somatic cell regulations can be maintained only in the diploid state in mammals. However, besides the above-mentioned centrosome loss, the exact cellular processes underlying the haploid intolerance in mammalian somatic cells are largely unknown.

A previous screening for compounds affecting haploid stability found 3-hydroxy-3-methylglutaryl-coA reductase inhibitors statins to facilitate the haploid-to-diploid conversion of HAP1 cells (9). Statin-mediated haploid destabilization occurred not through cholesterol depletion, a primary downstream target of statins, but through evoking endoplasmic reticulum (ER) stress (10). However, the pleiotropic effects of statins on broad cellular processes precluded further investigation of the link between ER homeostatic control and haploid instability in human cells. Therefore, molecular and cellular principles potentially destabilizing the haploid state under ER stress are entirely unknown. Clarifying these issues would elucidate key aspects of cell regulations that limit the sustainability of the haploid state in the somatic stage in the mammalian life cycle.

ER stress management is governed by unfolded protein response (UPR) (11). In mammals, UPR consists of three signaling pathway branches mediated by different ER transmembrane proteins, PKR like ER kinase (PERK)/eukaryotic translation initiation factor 2α kinase 3 (EIF2AK3), inositol requiring enzyme 1 (IRE1), and activating transcription factor 6 (ATF6) (12). In nonstressed conditions, these signaling transducers are sequestered in inactive states by direct interaction with an ER chaperone, binding immunoglobulin protein (BiP)/glucose-related protein 78 (Grp78)/heat shock protein family A member 5 (HSPA5) (13, 14, 15). Upon the accumulation of unfolded proteins in the ER lumen, pools of ER chaperones, including BiP, are recruited for chaperoning the unfolded proteins, resulting in the release and activation of these UPR transducers.

PERK is activated through dimerization and autophosphorylation. Active PERK then phosphorylates eukaryotic translation initiation factor 2A (eIF2α) to suppress general mRNA translation and reduce the burden of nascent protein folding (16). Meanwhile, phosphorylated eIF2α selectively increases translation of ATF4 (17), which in turn triggers increased transcription of numerous genes associated with ER stress management, including ER chaperones and autophagic factors (18, 19, 20). IRE1 is also activated through dimerization and autophosphorylation. Active IRE1 splices X-box-binding protein 1 (XBP1) mRNA, inducing the translation of the active form of XBP1 (21). XBP1 then mediates transcription of numerous genes involved in alleviating ER stress, such as ER chaperones and ER-associated degradation factors for clearance of misfolded protein (22). Activation of ATF6 is mediated through its relocation from ER to the Golgi apparatus, where it is cleaved by Golgi-resident site-1 and site-2 proteases (23, 24). PERK is also involved in the full activation of ATF6 (25). Then, the cleaved form of ATF6 released to the cytoplasm is translocated into the nucleus to mediate transcription of XBP1, chaperones, and ER-associated degradation factors (21, 26, 27, 28, 29). These reactions mediated by UPR pathways alleviate ER stress and promote cell survival.

However, when ER stress is prolonged or unmanageable, UPR changes its function to drive apoptosis and remove damaged cells. A proapoptotic transcription factor, C/EBP homologous protein (CHOP)/growth arrest and DNA damage inducible gene 153 (GADD153) is a main mediator of ER stress-induced apoptosis (30, 31, 32, 33), which is transcriptionally upregulated mainly by ATF4 and supportively by ATF6 (17, 26, 34). Therefore, the roles of UPR are highly cell context-dependent, but cellular conditions determining the balance between the prosurvival and proapoptotic function of UPR remain largely unknown (35, 36).

In this study, we found that haploid human somatic cells are significantly less efficient in solving ER stress and more prone to ER stress-induced apoptosis than isogenic diploid counterparts. Comparative biochemical analyses revealed that the induction of intermediate levels of ER stress through different stressors activated proapoptotic signaling preferentially in haploids. This ploidy-dependent alteration in UPR functionality drove the rapid expansion of coexisting diploid over haploid cell population, lowering the stability of the haploid state under ER stress. Interestingly, haploid cells suffered a higher level of proteotoxicity than diploids even in unperturbed conditions, alleviation of which by a chemical chaperone significantly resolved the innate instability of the haploid state. These findings indicate that haploidy-linked proneness to ER stress limits the proliferative capacity in the haploid state, creating a fundamental cause of the haploid instability in mammalian somatic cells.

## Results

### ER stress induction aggravates haploid instability through a haploidy-selective cell proliferation suppression

We previously found that statin impaired the stability of the haploid state by evoking ER stress in a human haploid model cell line, HAP1 (10). However, the pleiotropic effects of mevalonate pathway suppression by statin precluded further investigation of a possible relationship between ploidy status and ER homeostatic controls. To address this issue, we tested the effects of more specific ER stress inducer tunicamycin (an inhibitor of N-glycosylation) on the stability of the haploid state in HAP1 cells.

**Figure 1:**
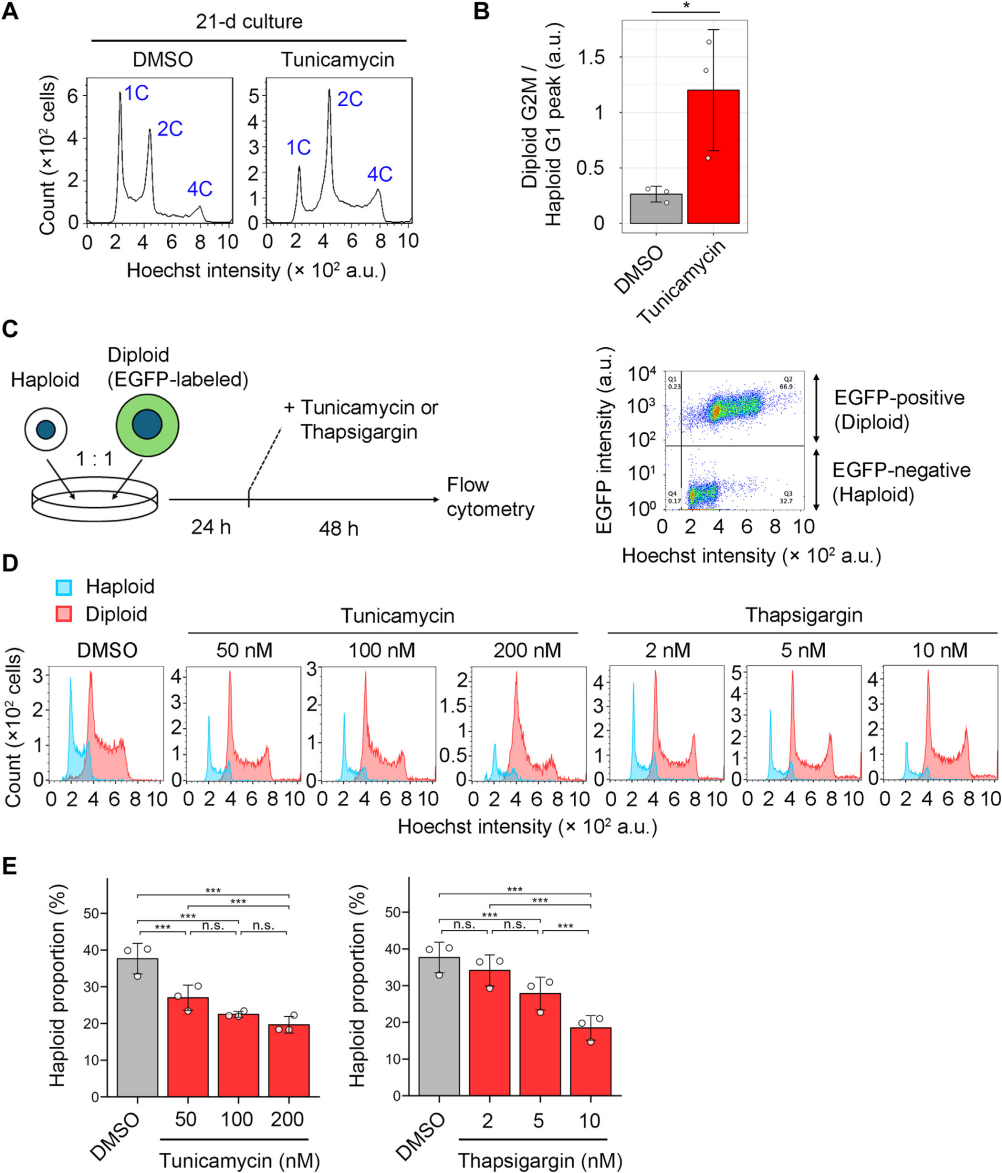
**ER stress induction causes destabilization of the haploid state in HAP1.***A*, flow cytometric analysis of DNA content in originally haploid HAP1 culture after consecutive passages in the absence or presence of 50 nM tunicamycin for 21 days. DNA was stained by Hoechst 33342. The labels on the plot (1C, 2C, and 4C) are relative DNA amounts (C-value) corresponding to haploid G1, haploid G2M/diploid G1, and diploid G2M, respectively. An example of DNA content analysis in the haploid cell culture before long-term passages (*i.e.*, day 0) is shown in Figure 1*A*. *B*, ratio of diploid G2M to haploid G1 populations in (*A*). Mean ± S.D. of three independent experiments (sampled at day 20, 21, or 23 of the consecutive culture). The *asterisk* indicates a statistically significant difference between conditions (∗*p* < 0.05, the Brunner-Munzel test). *C*, scheme of haploid-diploid coculture experiment. An example of a *dot plot* of EGFP intensity against the Hoechst signal in the coculture flow cytometric analysis is shown on the *right*. Cell populations originating from haploid or diploid cells were distinguished based on EGFP signal intensity. *D*, DNA content analysis of cocultured haploid and diploid cells treated with different concentrations of tunicamycin or thapsigargin for 48 h. Representative data from three independent experiments. *E*, the proportion of haploid cells in the haploid-diploid coculture. Mean ± S.D. of three independent experiments for each condition. *Asterisks* indicate statistically significant differences between conditions (n.s.: not significant, ∗∗∗*p* < 0.001, the Steel-Dwass test). For comparison, identical control data (DMSO) are shown in each graph in (*E*). DMSO, dimethylsulfoxide; ER, endoplasmic reticulum.

We first treated purified haploid cells with or without tunicamycin and compared the lifetime of the haploid population between the conditions during consecutive passages (Figs 1, *A* and *B*, and S1*A*). In the untreated condition, the initially pure haploid population gradually converted to diploids in successive passages for a few weeks, resulting in a reduction of haploid G1 proportion with the emergence of diploid G2/M proportion in flow cytometric DNA content analysis (Figs. 1, *A* and S1*A*). Treatment with 50 nM tunicamycin, which allowed cell proliferation with a detectable level of UPR (Fig. S1*B* and also Fig. S3), further destabilized the haploid state during the long-term culture (Fig. 1*A*). Ratio of diploid G2M to haploid G1 population became 4.5 times higher in tunicamycin-treated culture than in control after 20 days (Figs 1*B* and also 6*C* for more extended culture). Tunicamycin treatment did not cause tetraploidization (Figs. 1*A*, and also 6*C*), demonstrating that it specifically facilitated diploid cell expansion rather than causing general polyploidization. Tunicamycin at a higher concentration than 100 nM completely blocked cell proliferation, precluding the investigation of its effects on long-term haploid stability (Fig. S1*B*). We also attempted to test the effects of another ER stress inducer, thapsigargin (an inhibitor of intracellular Ca^2+^ transport), on the long-term stability of haploid cells. However, we could not keep cells proliferative with normal cell morphology for more than a few days in the presence of thapsigargin (Fig. S1*C*), precluding the long-term culture experiment.

Next, we sought to assess the possible deleterious effects of ER stress on the stability of the haploid state in a shorter period. For this, we conducted coculturing of haploid HAP1 cells with their isogenic diploids labeled with EGFP expression at a 1:1 ratio in the presence of different concentrations of tunicamycin and analyzed changes in haploid proportion after a 48-h incubation using flow cytometry (Fig. 1*C*). Even in untreated control, haploid proportion was reduced to 38% after 48 h, reflecting the less efficient proliferation of haploids than diploids (Fig. 1, *D* and *E*) (5). Tunicamycin significantly reduced haploid proportion compared to untreated control in a dose-dependent manner (Fig. 1, *D* and *E*). Importantly, the DNA content of the originally haploid population did not increase by tunicamycin treatment during the 48-h incubation (Fig. 1*D*). This result indicates that ER stress selectively suppresses haploid cell proliferation and helps expansion of diploidized population in originally haploid cell culture, rather than facilitating whole-genome duplication of haploid cells. A significant reduction in the haploid proportion in the coculture was also observed when treated with thapsigargin (Fig. 1, *D* and *E*). Therefore, the haploidy-selective antiproliferative effects of ER stress inducers were common across different modes of action. As this haploidy-linked sensitization to ER stress was novel, we further addressed the principle underlying this phenomenon.

### Haploidy-linked aggravation of apoptosis underlies the haploidy-selective proliferation suppression under ER stress

**Figure 2:**
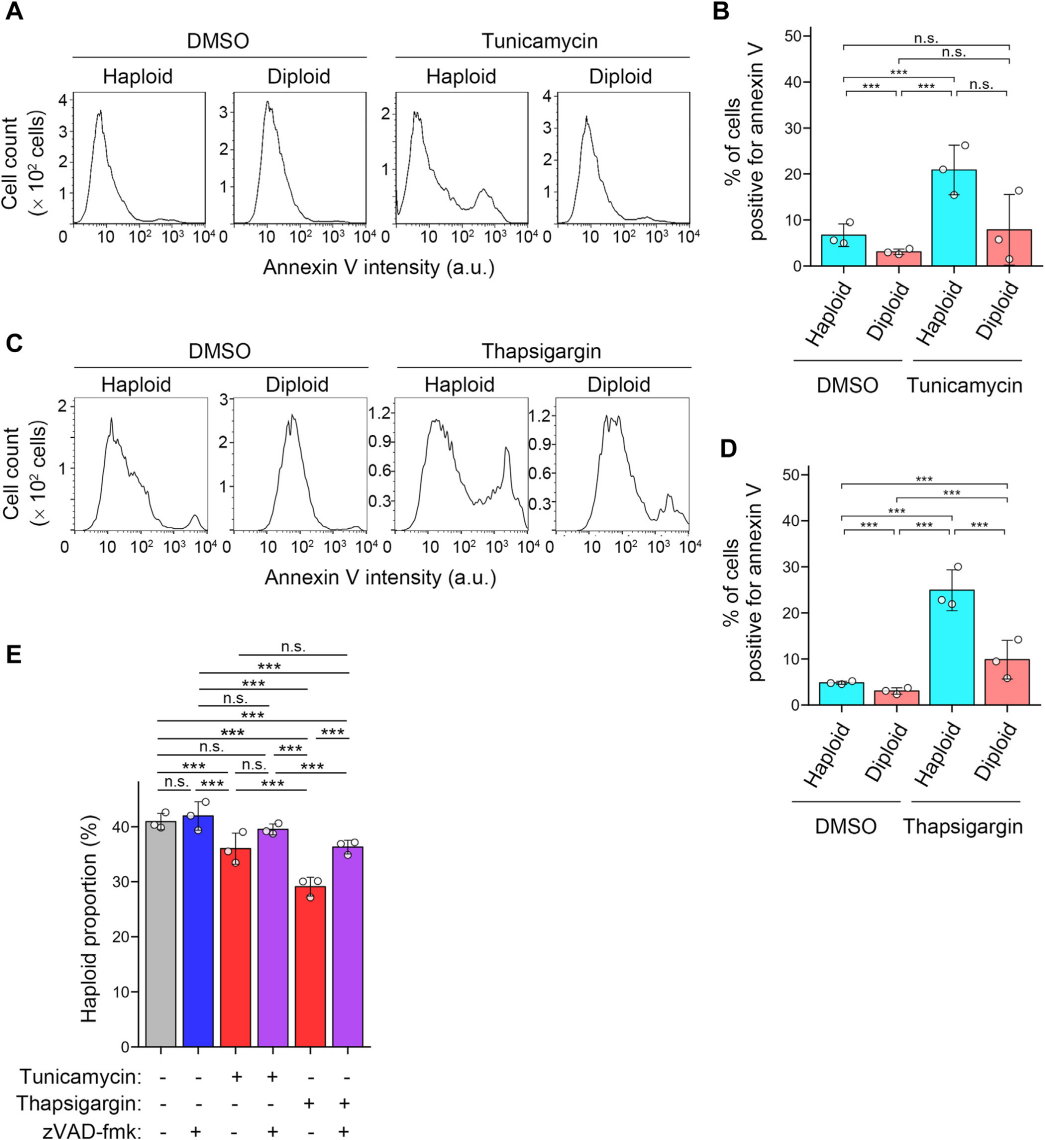
**Haploidy-linked aggravation of apoptosis under ER stress.***A*–*D*, flow cytometric analysis of annexin V-FITC staining in haploid or diploid HAP1 cells treated with or without 50 nM tunicamycin (*A* and *B*) or 10 nM thapsigargin (*C* and *D*) for 48 h. Histogram of annexin V-FITC intensity (*A* and *C*) and proportion of annexin V-FITC-positive cells (*B* and *D*). Mean ± S.D. of three independent experiments. *Asterisks* indicate statistically significant differences between ploidies (n.s.: not significant, ∗∗∗*p* < 0.001, the Steel-Dwass test). *E*, the proportion of haploid cells in coculture of unlabeled haploid and EGFP-labeled diploid cells treated by tunicamycin or thapsigargin with or without zVAD-fmk for 49 h. Mean ± S.D. of three independent experiments for each condition. *Asterisks* indicate a statistically significant difference among conditions (n.s.: not significant, ∗∗∗*p* < 0.001, the Steel-Dwass test). ER, endoplasmic reticulum.

We reasoned that ER stress inducers reduced haploid proportion in the haploid-diploid coculture through selective inhibition of cell cycle progression or induction of cell death. The DNA content analysis showed no difference in cell cycle distribution between haploids and diploids treated with ER stress inducers (Fig. 1*D*). Consistent with this, the overall trends of the changes in the expressions of cell cycle makers (including proliferating cell nuclear antigen, cyclin E, and cyclin A) were similar between haploids and diploids upon tunicamycin or thapsigargin treatment (Fig. S2, *A*–*D*). Therefore, we next conducted annexin V-FITC staining assay to compare the frequency of early apoptotic cells between haploid and diploid HAP1 cell cultures treated with 50 nM tunicamycin for 48 h (Fig. 2, *A* and *B*). In nontreated control, only a small cell proportion was annexin V-FITC positive in haploid and diploid cell cultures (6.9% and 3.0%, respectively; Fig. 2*B*), with a slightly higher frequency in haploids. The proportion of annexin V-FITC-positive cells increased by tunicamycin in haploids, while it remained at a low level in diploids (21% and 7.9% in haploids and diploids, respectively; Fig. 2*B*). The haploidy-linked aggravation of apoptosis was also observed upon thapsigargin treatment in the same assay (Fig. 2, *C* and *D*). Therefore, haploid cells were more prone to apoptosis compared to diploids under different types of ER stresses.

To test the causality between the haploidy-liked aggravation of apoptosis and the destabilization of the haploid state under ER stress, we tested the effects of a caspase inhibitor, zVAD-fmk, on haploid proportion in the haploid-diploid coculture treated with 50 nM tunicamycin or 10 nM thapsigargin (Fig. 2*E*; see Experimental procedures). zVAD-fmk restored the haploid proportion in tunicamycin- or thapsigargin-treated cocultures, demonstrating that apoptosis blockage alleviates the ploidy-dependent bias of cell proliferation under ER stress (Fig. 2*E*). These results suggest that the haploidy-linked aggravation of apoptosis is a primary reason of the haploid destabilization under ER stress.

### UPR is biased toward a proapoptotic mode in haploids under ER stress

**Figure 3:**
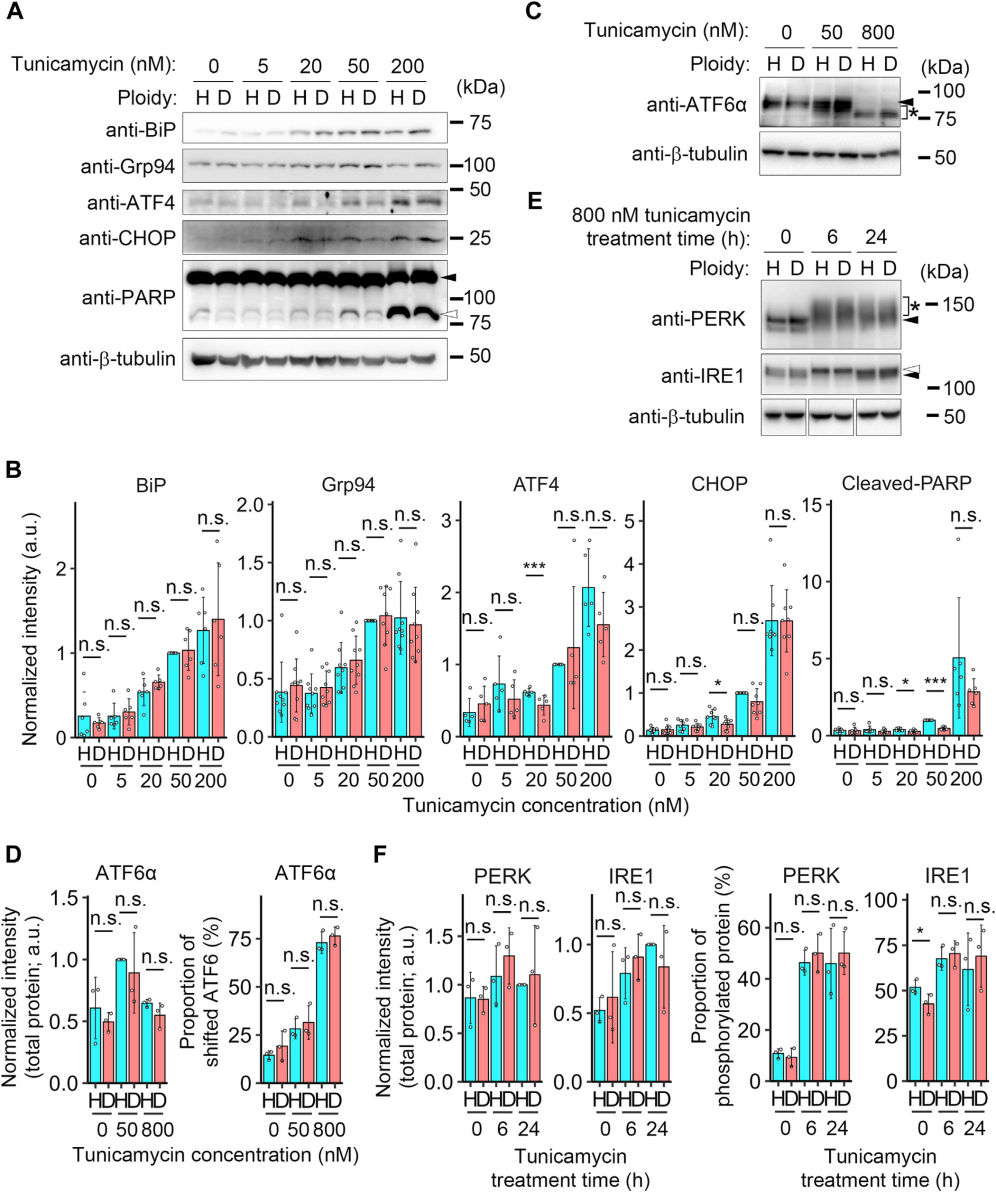
**Haploidy-linked upregulation of CHOP expression and PARP cleavage under ER stress.***A*, *C*, and *E*, immunoblotting of UPR components and proapoptotic factors in haploid or diploid HAP1 cells treated with different concentrations of tunicamycin for 24 h (*A* and *C*) or for 6 and 24 h (*E*). Unmodified molecular species are indicated by *closed arrowheads*. Modified species (cleaved PARP, deglycosylated ATF6α, or phosphorylated PERK or IRE1) are indicated by *open arrowheads* or *asterisks*. β-tubulin was detected as a loading control. Representative results from ≥3 independent experiments. *B*, quantification of the relative intensity of BiP, Grp94, ATF4, CHOP, or cleaved PARP. Protein loading differences were corrected based on β-tubulin signals. Mean ± S.D. of ≥5 independent experiments. *Asterisks* indicate statistically significant differences between ploidies (n.s.: not significant, ∗*p* < 0.05, ∗∗∗*p* < 0.001, the Brunner-Munzel test). *D* and *F*, *Left*: quantification of the relative intensity of the total amount of ATF6α (*D*) or PERK and IRE1 (*E*). Protein loading differences were corrected based on β-tubulin signals. *Right*: proportion of downshifted (deglycosylated) ATF6α (*D*) or phosphorylated PERK and IRE1 (*E*) to total protein. Mean ± S.D. of three independent experiments. *Asterisks* indicate statistically significant differences between ploidies (n.s.: not significant, ∗*p* < 0.05, the Brunner-Munzel test). ATF, activating transcription factor; BiP, binding immunoglobulin protein; CHOP, C/EBP homologous protein; ER, endoplasmic reticulum; IRE1, inositol requiring enzyme 1; PERK, PKR like ER kinase; UPR, unfolded protein response.

To specify the molecular basis of the haploidy-linked aggravation of apoptosis upon ER stress, we compared the expression and posttranslational modifications of different components of UPR between haploids and diploids treated with different concentrations of tunicamycin for 24 h using immunoblotting. Tunicamycin treatment induced UPR events, including increased expression of ER chaperones, BiP, and GRP94/HSP90B1/endoplasmin, and ATF4 in a dose-dependent manner both in haploids and diploids (Fig. 3, *A* and *B*). We did not observe consistent differences in the expression of these factors between haploids and diploids across conditions, except for a weak trend of lower expression of BiP in haploids than in diploids (Figs. 3*B* and also 4). We also compared the changes in ATF6α. Among multiple antibodies tested, only an antibody against C-terminal residues of ATF6α could reliably recognize the protein in our experiments (Figs. 3*C* and also S6*E*). For this reason, we could not detect an active cleaved form of ATF6α. However, using this antibody, stress-induced activation of ATF6α could be assessed by the changes in the total amount of the uncleaved form or stress-associated deglycosylation (Fig. 3, *C* and *D*) (37, 38, 39). Tunicamycin did not reduce the total amount of uncleaved ATF6α but induced drastic deglycosylation in a dose-dependent manner both in haploids and diploids (Fig. 3, *C* and *D*). The extent of deglycosylation of ATF6α upon tunicamycin treatment was equivalent between haploids and diploids (Fig. 3*D*). We also analyzed PERK and IRE1 phosphorylation, but the mobility shift of these UPR sensors was unclear in the samples treated with 0 to 200 nM tunicamycin for 24 h, precluding us from quantitatively assessing the effect of ploidy difference on their reactivities (Fig. S2*E*). Therefore, we tested PERK and IRE1 in 800 nM tunicamycin-treated cells, covering the earlier phase of UPR (6 h after tunicamycin administration; Fig. 3*E*). In this condition, both PERK and IRE1 became phosphorylated with a slight increase in total protein expression level (Fig. 3, *E* and *F*). However, the level of their expression or phosphorylation was equivalent between haploids and diploids (Fig. 3*F*). Therefore, many UPR components were equivalently reactive to tunicamycin-induced ER stress between the haploid and diploid states. The same trend of ploidy-neutral reactivities of UPR sensors and the haploidy-linked modest reduction in BiP expression was also observed in thapsigargin-treated samples (Fig. S3, *A*–*F*).

**Figure 4:**
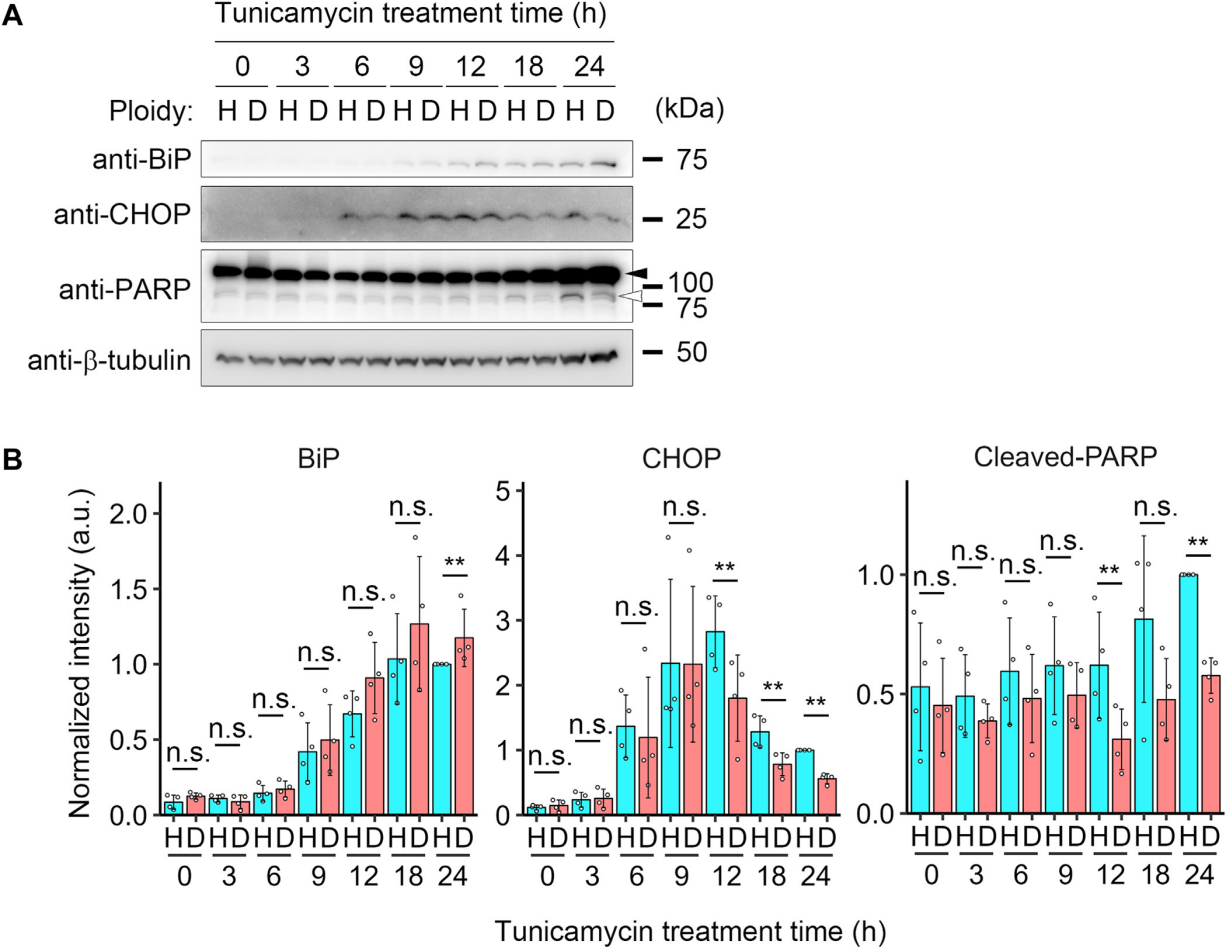
**Haploids are less efficient in resolving CHOP signaling upon acute ER stress.***A*, immunoblotting of BiP, CHOP, and PARP in haploid or diploid HAP1 cells treated with 50 nM tunicamycin for the indicated duration. The *closed* or *open arrowhead* indicates uncleaved or cleaved PARP, respectively. β-tubulin was detected as a loading control. Representative results from four independent experiments. *B*, quantification of the relative intensity of BiP, CHOP, or cleaved PARP. Protein loading differences were corrected based on β-tubulin signals. Mean ± S.D. of four independent experiments. *Asterisks* indicate statistically significant differences between ploidies (n.s.: not significant, ∗∗*p* < 0.01, the Brunner-Munzel test). BiP, binding immunoglobulin protein; CHOP, C/EBP homologous protein; ER, endoplasmic reticulum.

While we did not detect consistent ploidy-dependent differences in the UPR components mentioned above, we found a weak but consistent trend of the haploidy-linked substantiation of the increase in CHOP expression upon 24-h tunicamycin treatment either by immunoblotting or immunostaining (Figs. 3*B*, 4, and also S4, *A* and *B*). Immunostaining intensity of ATF4 positively correlated with that of CHOP either in haploids or diploids and significantly increased by tunicamycin in haploids but not in diploids (Fig. S4, *B* and C).

We also compared the extent of poly(ADP-ribose) polymerase (PARP) cleavage, a common consequence of proapoptotic signal activation under unsolved ER stress (40). Interestingly, haploids manifested a significantly higher magnitude of PARP cleavage than diploids in a low-concentration range of tunicamycin (Figs. 3*B* and also 4). Similar trends of the haploidy-linked aggravation of the proapoptotic signaling was also observed upon 24-h treatment with different concentrations of thapsigargin (Fig. S3, *A* and *B* and S5, *A* and *B*). Therefore, haploid cells were more prone to proapoptotic status than diploids upon UPR activation under moderate ER stress, consistent with the result in the annexin V binding assay (Fig. 2, *A*–*D*).

To obtain insight into the dynamics of the aggravation of proapoptotic signaling in haploid cells under ER stress, we next analyzed the time course of expressions of BiP, CHOP, and PARP cleavage during 24-h tunicamycin treatment in haploids and diploids (Fig. 4*A*). After 50 nM tunicamycin administration, the expression of BiP monotonically increased with similar kinetics between haploid and diploid cells, while we observed a weak trend of lower BiP level in haploids than in diploids throughout the time course (Fig. 4*B*). On the other hand, the expression of CHOP peaked around 9 to 12 h after tunicamycin administration and gradually decreased afterward until 24 h, both in haploids and diploids (Fig. 4, *A* and *B*). However, during 12 to 24 h, haploid cells kept a higher level of CHOP expression than diploids (Fig. 4*B*). Consistent with the prolonged CHOP expression, haploid cells manifested a substantially higher level of cleaved PARP than diploids during 12 to 24 h after tunicamycin administration (Fig. 4, *A* and *B*).

We also analyzed the time-course changes in BiP, CHOP, and PARP during 10 nM thapsigargin treatment (Fig. S5, *A* and *B*). By thapsigargin treatment, both BiP and CHOP upregulation peaked at an earlier time point than in the case of tunicamycin treatment. Consistent with the case of tunicamycin treatment, there was a weak trend of lowered BiP expression in haploids than in diploids throughout the thapsigargin treatment (Fig. S5*B*). In contrast to the case of tunicamycin treatment, diploids tended to express higher levels of CHOP than haploids during 6 to 18 h after administration of thapsigargin, when CHOP expression gradually decreased (Fig. S5*B*). Interestingly, however, this ploidy-dependent trend reversed at 24 h with a significantly higher level of CHOP remaining in haploids than in diploids (Fig. S5*B*), suggesting the prolonged CHOP expression in thapsigargin-treated haploids. Consistent with the case of tunicamycin treatment, cleaved PARP levels became higher in haploids than in diploids at 24 h after thapsigargin administration (Fig. S5*B*). Therefore, while UPR commenced and progressed with different dynamics between tunicamycin and thapsigargin treatment, haploids were commonly less efficient in adapting to and recovering from acute ER stress.

### Haploid cells suffer a higher level of protein aggregation either in the presence or absence of ER stressors

**Figure 5:**
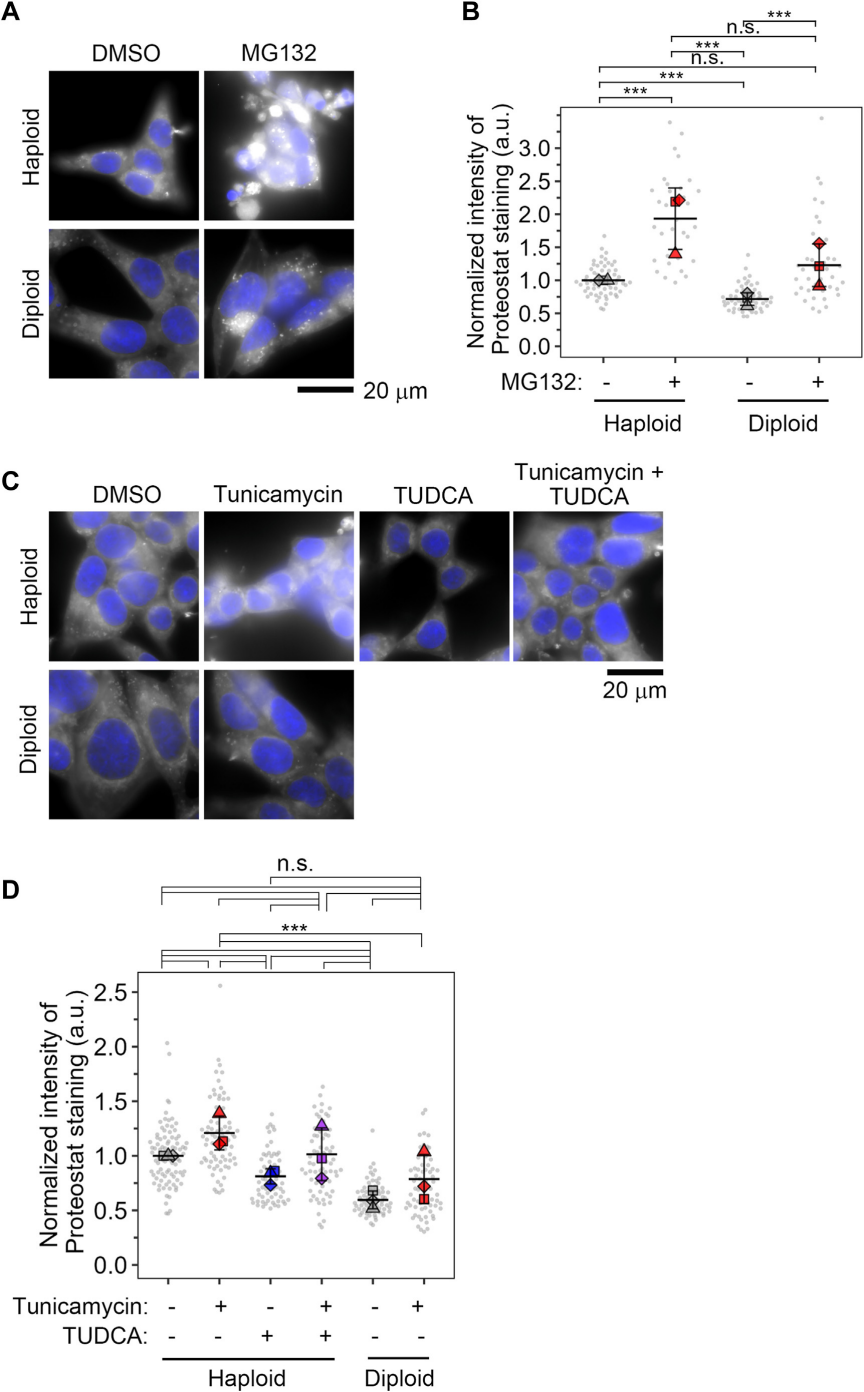
**Haploid cells suffer a higher level of protein aggregation.***A* and *C*, fluorescence microscopy of Proteostat dye staining in haploid or diploid HAP1 cells treated with or without 10 μM MG132 (*A*) or 50 nM tunicamycin in the presence or absence of 2.5 mM TUDCA (*C*). MG132 was treated for 12 h. Tunicamycin was treated for 24 h, and TUDCA was added at 12 h after the administration of tunicamycin. DNA was stained by Hoechst 33342 (shown in *blue*). *B* and *D*, quantification of cytoplasmic Proteostat signal in (*A* or *C*). Mean ± S.D. of three independent experiments (mean values within independent experiments are plotted as *squares*, *triangles*, or *diamonds*). *Asterisks* indicate a statistically significant difference among conditions (n.s.: not significant, ∗∗∗*p* < 0.001, the Steel-Dwass test). At least 32 or 63 cells (for *B* or *D*, respectively) were analyzed for each condition. Single-cell values are also plotted as small *gray circles*.

The above findings raised a possibility that haploid cells were less efficient in resolving ER stress, hence suffering prolonged stress status to induce proapoptotic signaling. To test this idea, we next assessed the extent of proteotoxicity in ER stress-induced haploids and diploids by postfixation cell staining using Proteostat dye that specifically labeled misfolded and aggregated proteins (41). We first tested the sensitivity of the dye by comparing its intensity in haploid and diploid cells treated with or without 10 μM MG132 (Fig. 5, *A* and *B*), a proteasome inhibitor expected to induce severe protein aggregation (41). Interestingly, Proteostat staining was significantly more intense in haploids than in diploids in the nonperturbed condition (Figs. 5, *B* and *D* and also S5*C*). MG132 treatment evidently increased Proteostat intensity both in haploid and diploid cells, confirming the protein misfolding detectability by the assay (Fig. 5*B*). The effect of MG132 was severer in haploids, resulting in significantly stronger Proteostat intensity in haploids than diploids in the presence of MG132 (Fig. 5*B*).

Next, we compared Proteostat intensity between haploid and diploid cells treated with or without 50 nM tunicamycin (Fig. 5, *C* and *D*), which specifically activated proapoptotic signaling in haploids (Fig. 3). In diploids, tunicamycin treatment did not significantly change Proteostat intensity (Fig. 5*D*). This result suggests that UPR upregulation observed in this condition (Figs. 3 and 4) sufficiently resolves proteotoxic state upon tunicamycin treatment in the diploid state. In contrast, Proteostat intensity significantly increased upon tunicamycin treatment in haploids (Fig. 5*D*). We also measured Proteostat intensity in 10 nM thapsigargin-treated cells (Fig. S5*C*). Whereas Proteostat intensity increased by thapsigargin in haploids and diploids, haploids showed significantly higher intensity than diploids (Fig. S5*C*). Therefore, haploids were less efficient in solving protein misfolding and aggregation under ER stress. Moreover, the finding that haploids manifested higher Proteostat intensity even in unperturbed conditions demonstrated the elevation of basal proteotoxicity in the haploid state compared to diploids.

### A chemical chaperone restores the stability of haploid cells treated either in the presence or absence of tunicamycin

**Figure 6:**
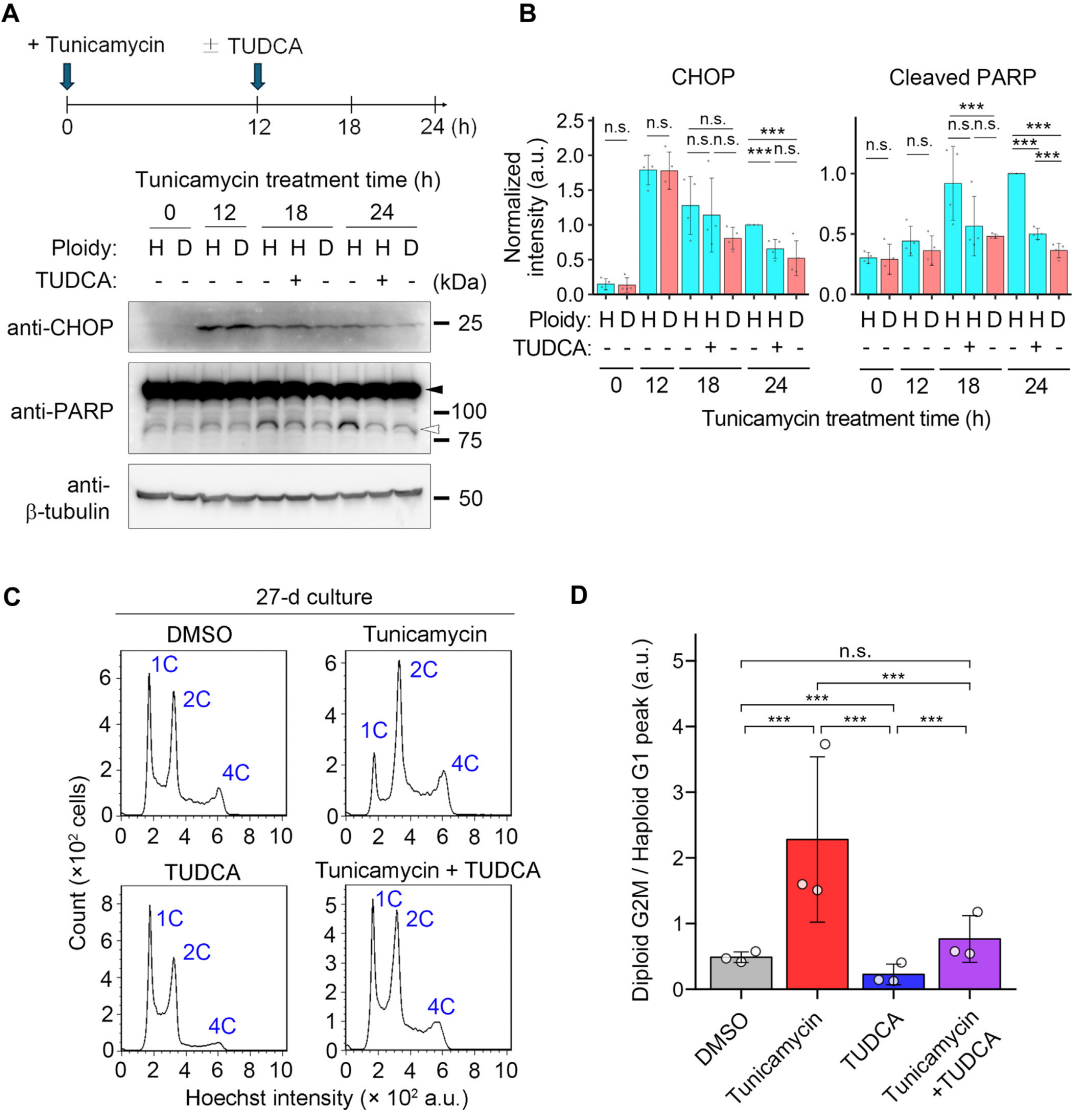
**TUDCA stabilizes the haploid state under ER stress.***A*, immunoblotting of CHOP and PARP in haploid or diploid HAP1 cells treated with 50 nM tunicamycin for the indicated duration. TUDCA was administrated to haploid cell culture at 12 h after the introduction of tunicamycin, as depicted in the scheme on top. The *closed* or *open arrowhead* indicates uncleaved or cleaved PARP, respectively. β-tubulin was detected as a loading control. Representative results from four independent experiments. *B*, quantification of the relative intensity of CHOP, or cleaved PARP, at each time point in (*A*). Protein loading differences were corrected based on β-tubulin signals. Mean ± S.D. of four independent experiments. *Asterisks* indicate statistically significant differences among samples (n.s.: not significant, ∗∗∗*p* < 0.001, the Brunner-Munzel test for 0- and 12-h samples, the Steel-Dwass test for 18- and 24-h samples). *C*, flow cytometric analysis of DNA content in originally haploid HAP1 culture after consecutive passages in the absence or presence of 50 nM tunicamycin or TUDCA for 27 days. DNA was stained by Hoechst 33342. An example of DNA content analysis in the haploid cell culture before long-term passages (*i.e.*, day 0) is shown in Figure 1*A*. *D*, ratio of diploid G2M to haploid G1 populations in (*C*). Mean ± S.D. of three independent experiments (sampled at day 27 of the consecutive culture). *Asterisks* indicate statistically significant differences among samples (n.s.: not significant, ∗∗∗*p* < 0.001, the Steel-Dwass test). CHOP, C/EBP homologous protein; ER, endoplasmic reticulum.

We next addressed the causality between the poorer ability to solve protein aggregation and proneness to proapoptotic signaling in haploid cells under ER stress. For this, we attempted to restore protein folding capacity in haploid cells by treating them with a chemical chaperone TUDCA and tested its effects on proapoptotic signaling in haploid cells. We introduced TUDCA in haploid cell culture at 12 h after administering 50 nM tunicamycin and tested the time course of CHOP expression and PARP cleavage (Fig. 6, *A* and *B*). Prior to this analysis, we confirmed that the introduction of TUDCA in this schedule reduced Proteostat signal to ∼80% in haploids either in the presence or absence of tunicamycin (Fig. 5*D*), showing that TUDCA was indeed effective in facilitating the resolution of either basal or artificially induced proteotoxic stress in the haploid state. Consistent with the results in Figure 3, CHOP expression peaked at 12 h posttunicamycin administration. However, TUDCA treatment from that time substantially suppressed CHOP expression at 24 h (Fig. 6*B*). Importantly, TUDCA also reduced PARP cleavage at 24 h in haploids to the level closer to diploids (Fig. 6*B*). Therefore, even when initial CHOP expression was fully induced, the later replenishment of chaperone function sufficiently canceled the haploidy-linked aggravation of proapoptotic signaling. These data indicate that the lower efficiency in ER stress alleviation is the main cause of proapoptotic signal activation in haploid cells. In contrast to the case of tunicamycin, TUDCA failed to alleviate protein aggregation in thapsigargin-treated haploids even when we coadministrated it with thapsigargin at the same time, precluding us from testing the effects of stress alleviation on proapoptotic signaling (Fig. S5*C*).

Next, we tested whether the replenishment of the chaperone function by TUDCA restored the stability of the haploid state in tunicamycin-treated long-term cell culture (Fig. 6, *C* and *D*). Consistent with the previous data (Fig. 1*A*), tunicamycin treatment accelerated the haploid-to-diploid conversion during 27-days culture. However, the acceleration of the haploid-to-diploid conversion was canceled by cotreating TUDCA with tunicamycin (Fig. 6, *C* and *D*). Moreover, we found that a single treatment of TUDCA significantly stabilized the haploid state during the long-term culture when compared to nontreated control (Fig. 6*D*). Considering that haploids suffered severe basal proteotoxic stress (Fig. 5, *B* and *D*), this result indicates that not only artificially induced ER stress but also basal ER stress in unperturbed haploid cells contributes to the instability of the haploid state in mammalian somatic cells.

### Effects of inhibition or suppression of UPR sensors on relative haploid proliferation

We reasoned that if any single UPR branch caused the haploid-selective proapoptotic signaling, specific suppression of the branch would restore relative haploid proliferation under ER stress. To address this idea, we tested the effects of inhibition of each UPR sensor by GSK2656157 (a PERK inhibitor), 4μ8C (an IRE1 inhibitor), or Ceapin-A7 (an ATF6α inhibitor) on haploid proportion in the haploid-diploid coculture after 48-h incubation in the presence or absence of 50 nM tunicamycin (Fig. S6, *A* and *B*). Among these inhibitors, only Ceapin-A7 significantly increased haploid proportion either in the presence or absence of tunicamycin, substantially reducing the tunicamycin-induced PARP cleavage in haploids (Fig. S6, *A*–*D*). However, RNAi-mediated ATF6α depletion did not restore haploid proportion in the tunicamycin-treated coculture, making the specificity of the effect of Ceapin-A7 questionable (Fig. S6, *E* and *F*; see Discussion). PERK depletion did not restore haploid proliferation either (Fig. S6*F*). We also tested the effects of the inhibitor of UPR sensors on thapsigargin-treated coculture, but none of the inhibitors restored the relative haploid proliferation in this condition (Fig. S6*G*). These data do not support the simple view that any single UPR branch alone makes haploids more prone to general ER stresses, suggesting the complicated nature of the molecular mechanisms governing the phenomenon.

## Discussion

### Destabilization of the haploid state under ER stress

The instability of the haploid state with gradual haploid-to-diploid conversion is commonly observed in mammalian somatic cells. Elucidating the principle of this haploid instability is important for understanding the fundamental restriction imposed on mammalian evolutions and for improving the utilities of haploid cell techniques in mammalian species. We previously found two contributors to haploid-to-diploid conversion: (i) spontaneous whole-genome duplication *via* centrosome loss that causes chromosome missegregation and (ii) poorer proliferation of haploid cells compared to diploidized ones (5, 42). However, the causes of the poorer proliferation of haploid cells remained largely unknown. In this study, we specified the difference in ER stress tolerance between haploid and diploid cells, which would provide insight into the principle of the ploidy-linked difference in cell proliferation efficiency and the unstable nature of the haploid state in mammalian somatic cells.

Artificial induction of ER stress facilitated haploid-to-diploid conversion in long-term culture experiments. Our results from the haploid-diploid coculture experiments suggest that destabilization of the haploid state upon ER stress induction is mainly attributed to the haploidy-selective suppression of cell proliferation (Fig. 1). We also found that haploids underwent apoptosis significantly more frequently than diploids under ER stress (Fig. 2), while there was no detectable difference in the effects of ER stress inducers on cell cycle distributions between haploids and diploids (Figs. 1 and S2). Moreover, artificial circumvention of apoptosis restored relative proliferative ability of haploids under ER stress (Fig. 2). These findings support the idea that the haploidy-linked aggravation of apoptosis is a primary cause of the selective removal of haploid population that leads to the destabilization of the haploid state under ER stress.

Upon the administration of ER stress inducers, haploid cells activated UPR with overall similar dynamics as diploids (Figs. 3, 4, S3, and S5). However, haploid cells tended to show reduced BiP expression and manifest sustained CHOP upregulation with higher PARP cleavage levels than diploids. These findings suggest that the UPR mechanism is more biased toward proapoptotic mode in haploid cells than diploids. Interestingly, haploid cells manifested significantly stronger Proteostat staining signals than diploids after the administration of ER stress inducers (Fig. 5), indicating that haploids are less efficient in solving proteotoxic status under ER stress. Since replenishment of chaperone function by TUDCA reduced proapoptotic signaling in tunicamycin-treated haploids to diploid level (Fig. 6), the haploidy-linked bias toward proapoptotic mode of UPR would be mainly attributed to the prolonged ER stress due to the inefficient proteostatic control in haploids.

The roles of each UPR branch in haploidy-selective proapoptotic signaling remain unclear. We observed the haploidy-linked substantiation of the tunicamycin-induced upregulation of the ATF4-CHOP axis (Figs. 3, 4, and S4), suggesting the possible involvement of the PERK branch in the signaling. However, either pharmacological inhibition or RNAi-mediated depletion of PERK did not restore relative haploid cell proliferation in the haploid-diploid coculture under tunicamycin-induced ER stress (Fig. S6). While an ATF6α inhibitor Ceapin-A7 improved the relative haploid cell proliferation under tunicamycin-induced ER stress, this restoring effect was not reproduced by RNAi-mediated ATF6α depletion. In this connection, a recent study has revealed that ATF6α inhibition by Ceapin-A7 and RNAi-mediated ATF6α depletion caused substantially different patterns of changes in transcriptome, indicating possible nonspecific effects of the inhibitor (43). Therefore, it is possible that some nonspecific targets of Ceapin-A7 contribute to the haploidy-linked aggravation of the proapoptotic signaling under ER stress, and it is intriguing to elucidate such targets in future studies for understanding the molecular mechanisms of haploidy-linked proneness to ER stress. It is also important to note that Ceapin-A7 did not restore haploid cell proliferation under thapsigargin-induced ER stress, suggesting context-dependent differences in the mechanisms of haploidy-selective cell proliferation suppression. Overall, our data negate the simplified idea that any single UPR branch alone is responsible for the haploidy-selective proapoptotic regulation. Further study in the future, including comprehensive gene regulatory analyses using transcriptome information, would be required to understand how ER stress response signaling is altered to sensitize the proapoptotic pathway in haploid cells.

### Potential contributions of basal ER stress to haploid instability

It is important to note that haploid cells manifested a higher level of protein aggregation than diploids, even in an unperturbed condition (Fig. 5). We found that TUDCA substantially alleviated the basal protein aggregation and improved the long-term stability of the haploid state compared to the nontreated condition (Fig. 6). These findings indicate an interesting possibility that haploid cells are prone to naturally occurring ER stress that limits the proliferative capacity in the haploid state, creating a fundamental cause of the haploid instability in mammalian somatic cells. Related to this idea, a previous study has reported that deletion of HAC1 gene (a yeast orthologue of XBP1) results in the destabilization of an otherwise stable haploid state in budding yeasts (44), indicating that ER homeostasis is a crucial determinant of sustainability of somatic haploidy in broad species.

Since the basal level of ER stress-driven proapoptotic signaling was too low to be reliably detected by currently available methods, it was impossible to compare it between haploids and diploids accurately. We expect that molecular mechanisms and physiological effects of the basal ER stress-driven haploid cell suppression may be better addressed in tissue environments with a higher level of basal ER stresses. It would be particularly intriguing to address in the future how the ER stresses that take place in association with developmental or tumorigenic processes affect cellular behavior and tissue functionality in haploid embryogenesis or haploid cancer progression.

### Why are haploid cells less resistant to ER stress?

The reason for the proneness of haploid cells to ER stress and proteotoxicity remains unclear. A possible explanation may be the smaller cell size of haploids. In our previous estimation, haploid HAP1 cells were about half in volume compared to diploids (5). This theoretically results in about a 1.25-time increase in cell surface-to-volume ratio in haploids compared to diploids, potentially increasing the burden of membrane or secreted protein production on haploid cells. Interestingly, previous studies have reported haploidy-linked alterations in the transcription of genes encoding glycoproteins (7, 45). The potentially higher demand for ER productivity may cause more frequent protein misfolding in the basal cell state and lead to severe damage upon perturbations of ER functionality in haploid cells. Alternatively, cell size reduction may limit the capacity of ER to manage stresses by limiting its size through organelle size scaling mechanisms (46). Upon UPR activation under ER stress, cells increase their capacity to manage stresses by expanding the ER lumen in addition to enhancing its chaperone activities (47, 48, 49, 50). Cell size reduction may limit space for ER to expand upon ER stress and thus decrease the capacity to resolve protein misfolding in haploid cells. Since we previously found that thymidine-mediated transient cell cycle arrest arrowed excess cell growth and size increase in haploid HAP1 cells (42), we attempted to test its effects on proapoptotic signaling in haploids under ER stress. However, the transient cell cycle arrest *per se* activated proapoptotic signaling, precluding the investigation of the impacts of cell size increase on resolution of ER stress-induced proapoptotic signaling. To overcome this technical limitation, more sophisticated approaches to modulate cell size without affecting other cellular processes must be established in future studies. A comparative ultrastructural analysis of ER in the presence or absence of ER stress in haploid and diploid cells would also provide important insight into this issue in the future.

Besides cell size reduction, potential “haploinsufficiency (insufficient gene dosage with only single functional allele)” of critical stress-managing factors may attenuate the adaptive function of the UPR and limit the tolerability of haploid cells to ER stress. Consistent with this idea, dosage reduction or increase in specific genes have been demonstrated to alter cellular response to ER stress (51, 52). A weak but consistent trend of the haploidy-linked reduction in BiP expression (Figs. 3 and 4) may reflect the functional insufficiency in the mechanism governing the stress adaptive reactions. Future studies on the possible influence of haploinsufficiency in critical UPR factors would provide mechanistic insights into the aggravation of cell proliferation suppression in haploid cells.

### An integrated model of instability of the haploid state in mammalian somatic cells

**Figure 7:**
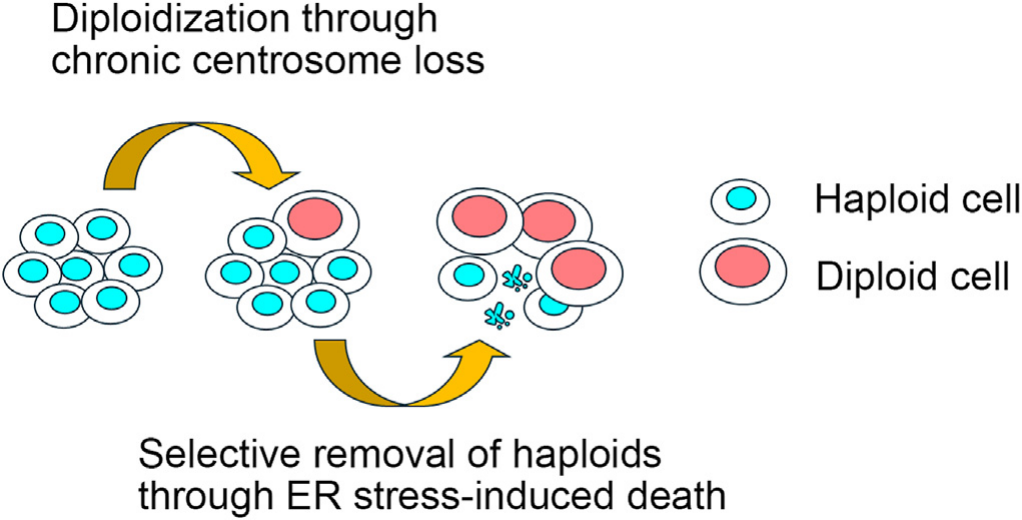
**An integrated model of the haploid instability in mammalian cells.** While chronic centrosome loss in a small population of haploids constantly produces the diploidized subpopulation, haploidy-linked susceptibility to ER stress selectively removes the haploid subpopulation and promotes the haploid-to-diploid conversion during the successive culture. ER, endoplasmic reticulum.

Based on our previous finding of centrosome loss and current finding of susceptibility to ER stress in haploid cells, we propose the following model of haploid instability (Fig. 7): Centrosome loss chronically occurring in a small proportion of haploids constantly causes mitotic spindle monopolarization defect often resulting in whole-genome duplication in the originally haploid cell culture (5). This gradually increases the diploidized cell subpopulation in the culture. In this haploid-diploid mixed culture, the innate susceptibility of haploids to ER stress leads to the gradual reduction of haploid proportion, further promoting the haploid-to-diploid conversion during successive cultures. Consistent with this view, pharmacological induction of centrosome loss facilitates nearly complete haploid-to-diploid conversion within 4 days (5), while the facilitation of the conversion by ER stress induction is much more gradual and takes a few weeks (Fig. 1*A*). Importantly, interventions to either process could improve the stability of haploid human cells (Fig. 6*D*) (42). Therefore, it is intriguing to address in the future whether combinatory control of these processes has more profound effects on stabilizing the haploid state in human cells.

The instability of the haploid state in the multicellular stage in mammals is a fundamental problem in the evolution of animal life cycles. At the same time, this is an important limitation of the application of mammalian haploid cell resources for genetics and bioengineering (53). Our new findings would provide a clue to understanding the principle of haploid intolerance in animals and modulating the stability of mammalian somatic haploidy to increase its utility in life science research fields.

## Experimental procedures

### Cell culture

Haploid cells (RRID: CVCL_Y019; from Haplogen GmbH) (54) were cultured in Iscove’s modified Dulbecco’s medium (Wako Pure Chemical Industries) supplemented with 10% fetal bovine serum (FBS) and 1× antibiotic-antimycotic solution (Sigma-Aldrich). Maintenance of haploid HAP1 cell populations using size-based cell sorting and establishment of diploid HAP1 cells were conducted as described previously (5).

For long-term cell passage experiments, freshly purified haploid HAP1 cells were cultured in the presence or absence of compounds described elsewhere. Cells were typically passaged every 2 days with replenishment of the compounds until they were subjected to flow cytometric analyses.

### Compounds and antibodies

Compounds were purchased from the distributors as follows. Tunicamycin: Cell Signaling Technology or Cayman Chemical. Thapsigargin: Wako. TUDCA: Tokyo Chemical Industry Co, Ltd GSK2656157, 4μ8C, and Ceapin-A7: Sigma-Aldrich. Z-Val-Ala-Asp(OMe)-CH_2_F (zVAD-fmk): Peptide Institute, Inc. Antibodies used in this study are listed in Table S1. The siRNA for mock depletion control is 5′-CGUACGCGGAAUACUUCGAtt-3′ (luciferase; DNA is shown in lowercase). siRNAs targeting ATF6α or PERK were purchased from Santa Cruz Biotechnology (sc-37699 or sc-36213, respectively). siRNA transfection was performed using Lipofectamine RNAiMAX (Thermo Fisher Scientific).

### Flow cytometry and cell sorting

Flow cytometric analyses and cell sorting were performed using a JSAN desktop cell sorter (Bay Biosciences). For DNA content analyses, cells were trypsinized using 0.05% trypsin-EDTA solution (Wako) and stained with 10 μg/ml Hoechst 33342 (Dojindo) for 15 min at 37 °C. For Annexin V assays, cells were suspended by treating with Accutase solution (PromoCell) and stained with Hoechst and 1 μg/ml Annexin V-FITC (Medical and Biological Laboratories) in binding buffer (10 mM Hepes, pH 7.4, 140 mM NaCl, and 2.5 mM CaCl_2_) for 15 min at 37 °C. The stained cells were filtered through a cell strainer and applied to the cell sorter. Data analyses were conducted using FlowJo software (BD Sciences; https://www.flowjo.com).

### Coculture experiment

Nonlabeled haploid and EGFP-expressing diploid cells (5 × 10^4^ cells/ml each) were mixed in a 1:1 ratio, 2 ml seeded on 6-well plates coated with collagen type I (Corning). After 24 h, ER stress inducers or UPR inhibitors were treated in the coculture. Approximately 48 h after the addition of the compounds, cells were subjected to flow cytometric DNA content analysis. For blocking caspase activities, 20 μM zVAD-fmk was administrated to cell cultures 1 h before the administration of ER stressors. zVAD-fmk was newly added every 24 h. When conducting RNAi, cells were seeded at 1.25 × 10^4^ cells/ml each, subjected to siRNA transfection after 24 h, and then treated with ER stress inducers after 24 h. The two mixed cell populations were separately counted based on the EGFP fluorescence signal.

### Immunoblotting and quantification

Cells were lysed with RIPA buffer (50 mM Tris–HCl, pH 7.6, 150 mM NaCl, 10 mM NaF, 10 mM β-glycerophosphate, 1% NP-40, 0.5% sodium deoxycholate and 0.1% SDS, protein inhibitor cocktail (cOmplete, Roche)) for 10 to 15 min on ice then clarified by centrifugation for 10 min with 15,000 rpm. The clarified lysate was mixed with 4 or 5× SDS-PAGE sample buffer, boiled for 5 or 10 min, and subjected to SDS-PAGE. Separated proteins were transferred onto an Immun-Blot polyvinylidene fluoride membrane (Bio-Rad). The blotted membranes were blocked with 0.3% skim milk in TTBS (50 mM Tris, 138 mM NaCl, 2.7 mM KCl, and 0.1% Tween 20), incubated with primary antibodies for 2 h or overnight at 4 °C, and incubated with horseradish peroxidase-conjugated secondary antibodies for 2 h at 25 °C or overnight at 4 °C. Each step was followed by three washes with TTBS. For IRE-1 (in Fig. 3) and CHOP (in Fig. S6) detection, antibodies were diluted with Can Get Signal Immunoreaction Enhancer Solution (Toyobo). The EzWestLumi plus ECL Substrate (ATTO) and a LuminoGraph II chemiluminescent imaging system (ATTO) were used for signal detection. Signal quantification with membrane background subtraction was performed using the Gels tool of ImageJ (NIH, https://imagej.net/ij/). Then, protein loading differences were corrected based on β-tubulin loading control signals.

### Immunofluorescence, microscopic observations, and quantification

Cells were fixed with 3.7% paraformaldehyde (Merck) in PBS) at room temperature for 10 min, followed by permeabilization with ice-cold 0.5% Triton-X100 in PBS containing 0.1 M glycine (Wako) for 5 min on ice. Fixed cells were treated with PBS containing 3% FBS (Gibco) and 3% bovine serum albumin (Wako) for 1 h on ice, incubated with primary antibodies >24 h at 4 °C, and with fluorescence-conjugated secondaries >24 h at 4 °C at indicated dilutions in PBS containing 5% FBS. DNA was stained with 0.5 μg/ml 4′,6-diamidino-2-phenylindole (DAPI, Dojindo). Following each treatment, cells were washed three times with PBS.

For detecting protein aggregations, cells were fixed with 4% paraformaldehyde in PBS at 25 °C for 30 min, followed by permeabilization with PBS containing 0.5% triton-X100 and 3 mM EDTA (pH 8.0) on ice with gentle shaking for 30 min, and incubated with 1× dual detection reagent in the Proteostat aggresome detection kit (containing Proteostat aggresome detection reagent and Hoechst 33342; Enzo Life Sciences) at 25 °C for 30 min. Following each treatment, cells were washed one or two times with PBS.

The stained cells were observed under a TE2000 microscope (Nikon) equipped with a ×60 1.4 NA Plan-Apochromatic, a CSU-X1 confocal unit (Yokogawa), and an iXon3 electron multiplier-charge coupled device camera (Andor) or ORCA-ER CCD camera (Hamamatsu Photonics), or with a Ti2 microscope (Nikon) with ×60 1.4 NA Apochromatic and Zyla4.2 sCMOS camera (Andor). Image acquisition was controlled by μManager (Open Imaging).

For quantification of ATF4 and CHOP signals within nuclei, nuclear areas were automatically segmented based on 4′,6-diamidino-2-phenylindole staining signals using a binary fill hole watershed in ImageJ. Then, mean values of ATF4 and CHOP immunostaining signals were measured within each nuclear area, followed by subtraction of background signals in noncell areas. For quantification of Proteostat staining, cytoplasmic areas in individual cells were manually segmented using the segmented line tool in ImageJ. Then, the mean value of Proteostat signal was measured within each cytoplasmic area, followed by the subtraction of background signals in noncell areas.

### Colorimetric cell proliferation assay

For cell viability assay, cells were seeded on 96-well plates at 9000 cells/well. After 24 h, cells were treated with different concentrations of tunicamycin. Forty-four hours after the addition of tunicamycin, 5% Cell Counting Kit-8 (Dojindo) was added to the culture, incubated for 4 h, and absorbance at 450 nm was measured using the Sunrise plate reader (Tecan).

### Statistical analysis

To compare two groups of categorical data or continuous numerical data not assumed to have normal distributions, we used the Brunner-Munzel test. To compare more than two groups of categorical data or continuous numerical data not assumed to have normal distributions, we used the Steel-Dwass test. We used the Steel test to compare a common control with each of multiple samples of categorical data. Statistical significance was set at *p* < 0.05 for all analyses. All statistical analyses were conducted with R software (4.2.1, https://www.r-project.org).

## Data availability

The all datasets supporting this study's findings are contained within this manuscript.

## Supporting information

This article contains supporting information.

## Conflict of interest

The authors declare that they have no conflicts of interest with the contents of this article.
