# Supplementary material for: Fragility of ER homeostatic regulation underlies haploid instability in human somatic cells
Source: J Biol Chem. 2024 Oct 19;300(11):107909. doi: 10.1016/j.jbc.2024.107909 (PMC11609373; doi:10.1016/j.jbc.2024.107909)
Supplement: Supporting Information [file mmc1.pdf]

Supporting Information:

Fragility of ER homeostatic regulation underlies haploid instability in human somatic cells

Sumire Ishida-Ishihara, Kan Yaguchi, Sena Miura, Ryoto Nomura, QiJiao Wang, Koya Yoshizawa, Kimino Sato, Guang Yang, Krisztina Veszelyi, Gabor Banhegyi, Eva Margittai, and Ryota Uehara

Materials included:

- Supplementary figure 1 (Figure S1) ⋯ S-1, S-2
- Supplementary figure 2 (Figure S2) ⋯ S-3, S-4
- Supplementary figure 3 (Figure S3) ⋯ S-5, S-6
- Supplementary figure 4 (Figure S4) ⋯ S-7, S-8
- Supplementary figure 5 (Figure S5) ⋯ S-9, S-10
- Supplementary figure 6 (Figure S6) ⋯ S-11, S-12
- Supplementary Table 1 (Table S1) ⋯ S-13

Supplementary figure 1

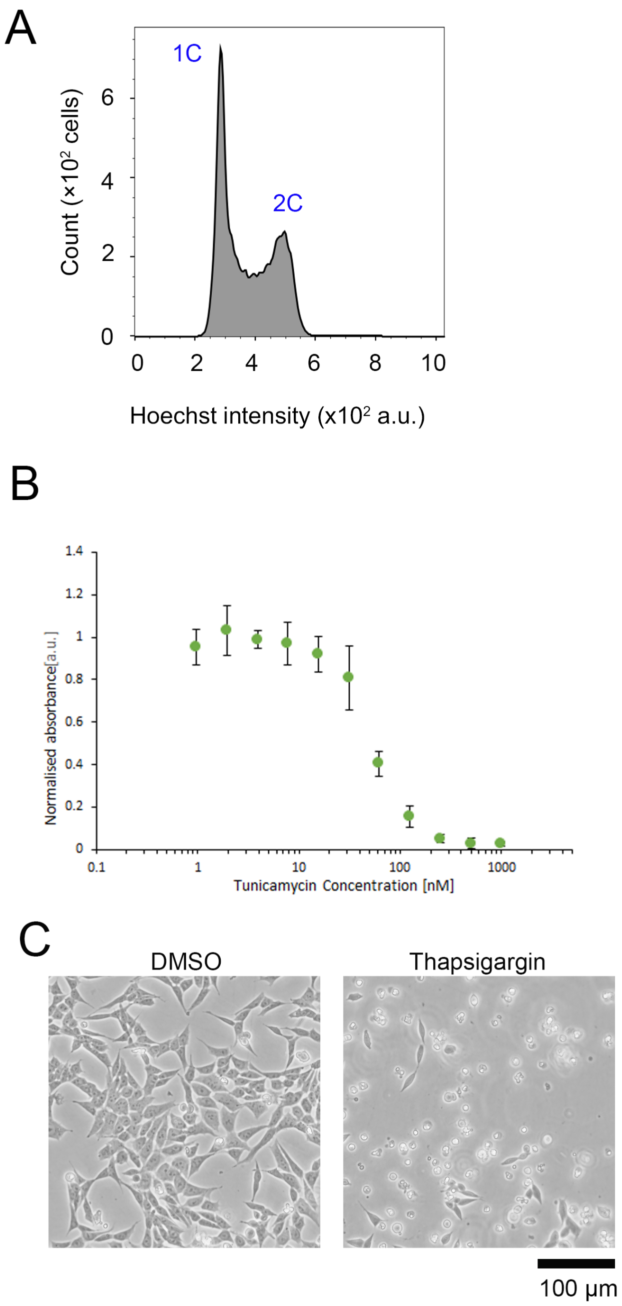

Supplementary figure 1

(A) Flow cytometric analysis of DNA content in the haploid cell culture at 20 h after thawing. Representative data from 2 independent experiments. **(B)** Dose-response curves of normalized absorbance in a colorimetric cell proliferation assay in haploid HAP1 cells treated with tunicamycin. Mean ± S.D. of 6 replicates from 3 independent experiments. **(C)** Microscopy of haploid HAP1 cells treated with or without 10 nM thapsigargin for 4 d. Representative data from 3 independent experiments.

Supplementary figure 2

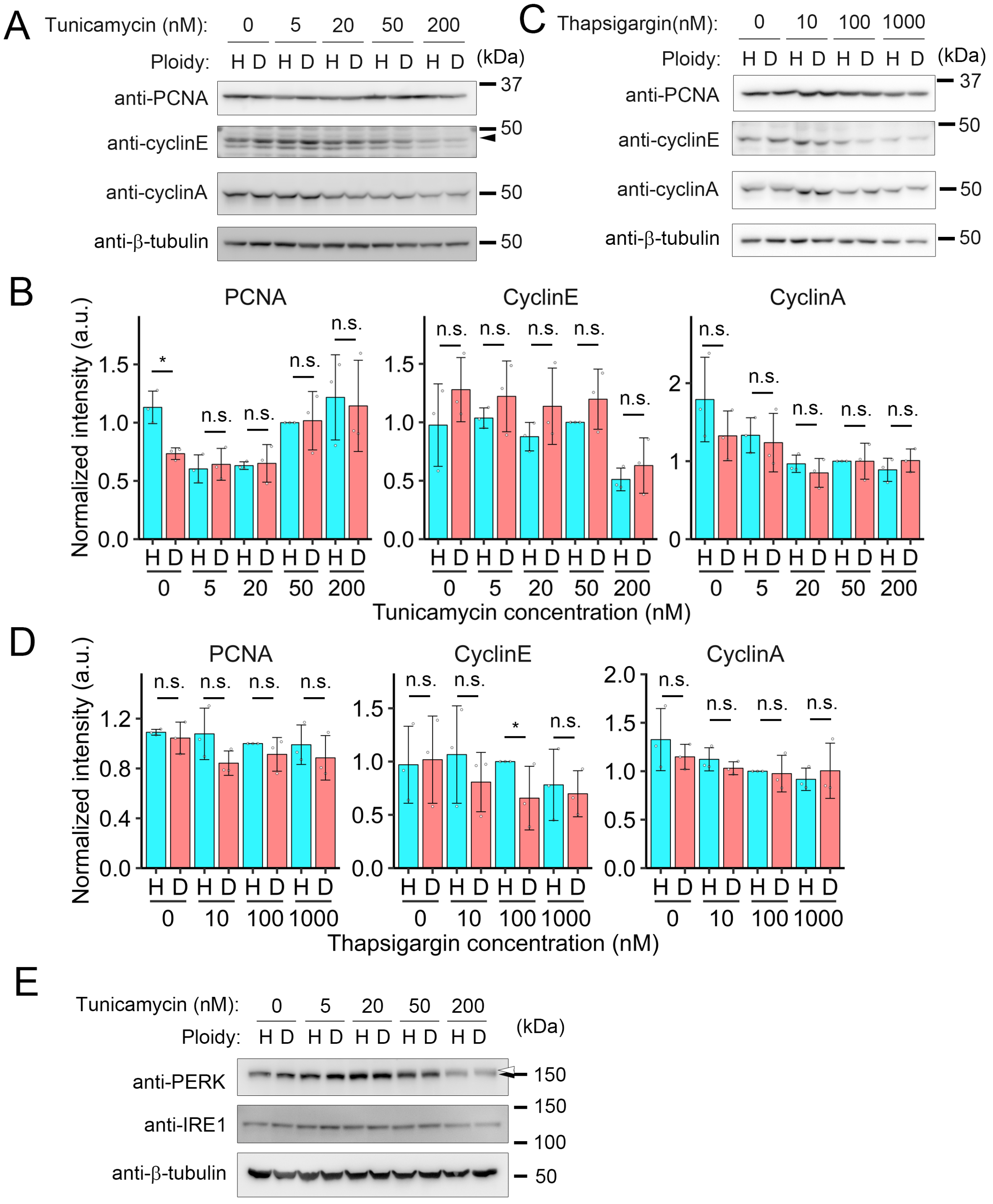

Supplementary figure 2

(A, C) Immunoblotting of PCNA, cyclin E, and cyclin A in haploid or diploid HAP1 cells treated with different concentrations of tunicamycin (A) or thapsigargin (C) for 24 h. β-tubulin was detected as a loading control. Representative results from 3 independent experiments. **(B, D)** Quantification of the relative intensity of PCNA, cyclin E, and cyclin A. Protein loading differences were corrected based on β-tubulin signals. Mean ± S.D. of 3 independent experiments. Asterisks indicate statistically significant differences between ploidies (n.s.: not significant, $^{*}p < 0.05$, the Brunner-Munzel test). **(E)** Immunoblotting of PERK and IRE1 in haploid or diploid HAP1 cells treated with different concentrations of tunicamycin for 24 h. β-tubulin was detected as a loading control. The closed or open arrowhead indicates unphosphorylated or phosphorylated PERK, respectively. Representative results from ≥3 independent experiments.

Supplementary figure 3

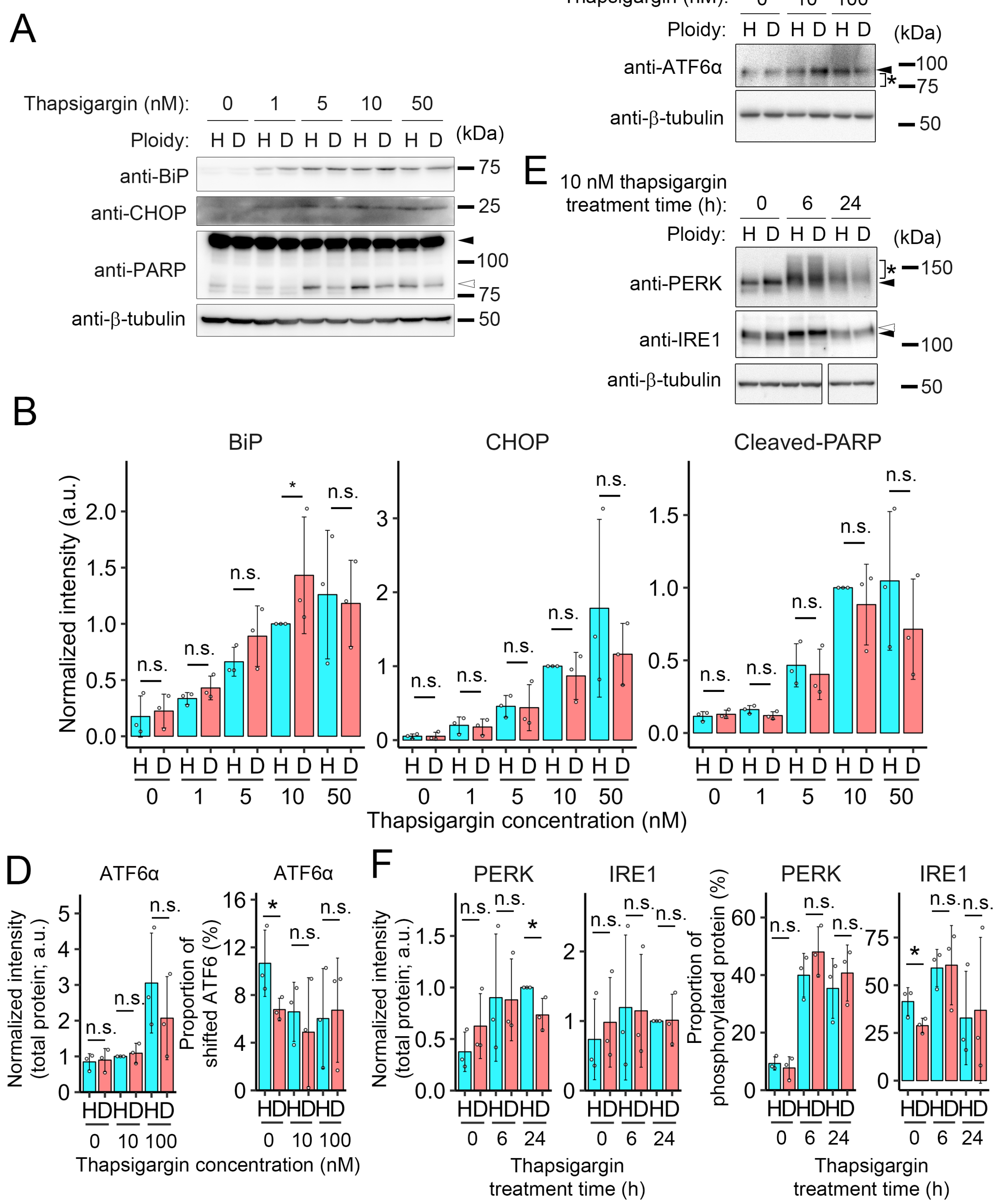

Supplementary figure 3

(A, C, E) Immunoblotting of UPR components and proapoptotic factors in haploid or diploid HAP1 cells treated with different concentrations of thapsigargin for 24 h (A and C) or for 6 h and 24 h (E). Unmodified molecular species are indicated by closed arrowheads. Modified species (cleaved PARP, deglycosylated ATF6α, or phosphorylated PERK or IRE1) are indicated by open arrowheads or asterisks. β-tubulin was detected as a loading control. Representative results from 3 independent experiments. **(B)** Quantification of the relative amount of BiP, CHOP, or cleaved PARP. Protein loading differences were corrected based on β-tubulin signals. Mean ± S.D. of 3 independent experiments. Asterisks indicate statistically significant differences between ploidies (n.s.: not significant, $^{*}p < 0.05$, the Brunner-Munzel test). **(D, F)** Left: Quantification of the relative intensity of the total amount of ATF6α (D) or PERK and IRE1 (E). Protein loading differences were corrected based on β-tubulin signals. Right: Proportion of downshifted (deglycosylated) ATF6α (D) or phosphorylated PERK and IRE1 (E) to total protein. Mean ± S.D. of 3 independent experiments. Asterisks indicate statistically significant differences between ploidies (n.s.: not significant, $^{*}p < 0.05$, the Brunner-Munzel test).

Supplementary figure 4

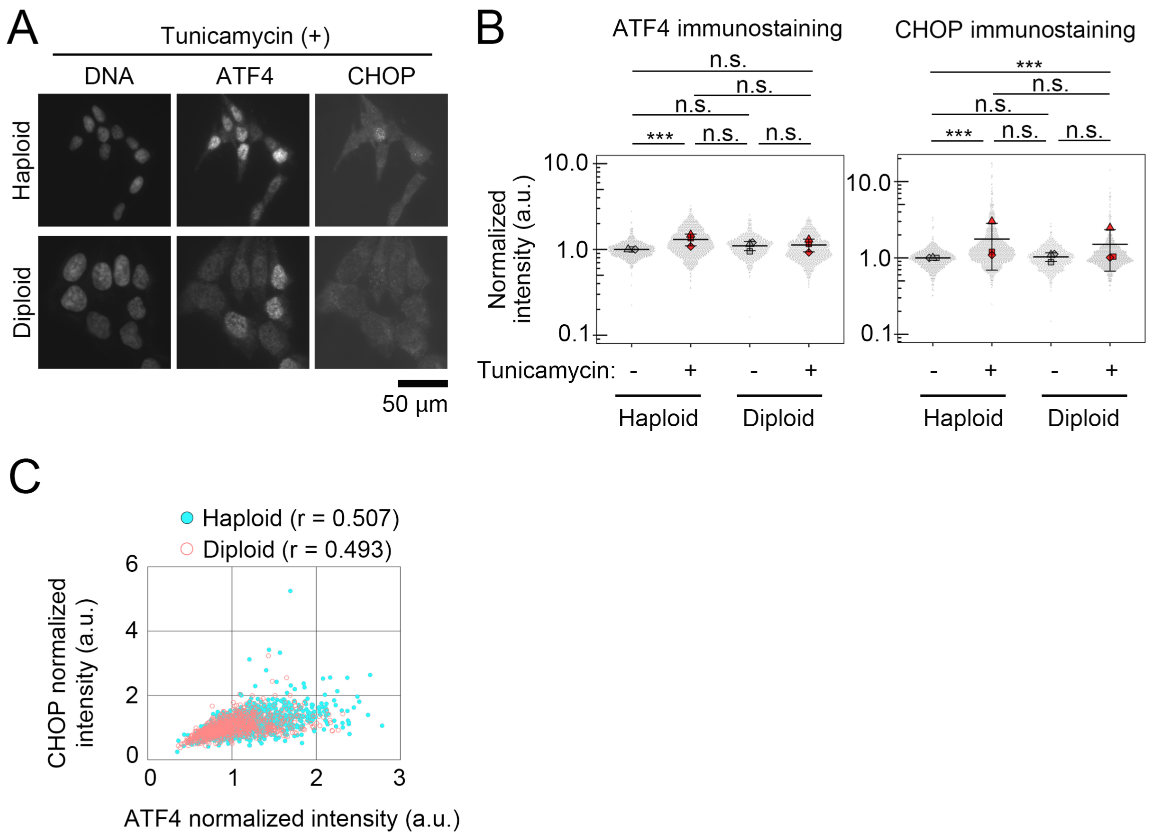

Supplementary figure 4

(A) Immunofluorescence microscopy of ATF4 and CHOP in haploid or diploid HAP1 cells treated with 50 nM tunicamycin for 24 h. DNA was stained by DAPI. **(B)** Quantification of ATF4 or CHOP signal at nuclei in A. Mean ± S.D. of ≥698 cells from 3 independent experiments. Asterisks indicate statistically significant differences among samples (n.s.: not significant, $^{***}p < 0.001$, the Steel-Dwass test). **(C)** A dot plot of CHOP signal against ATF4 signal in the immunostaining of haploids and diploids treated with tunicamycin for 24 h with correlation coefficient. Data are identical to those in B.

Supplementary figure 5

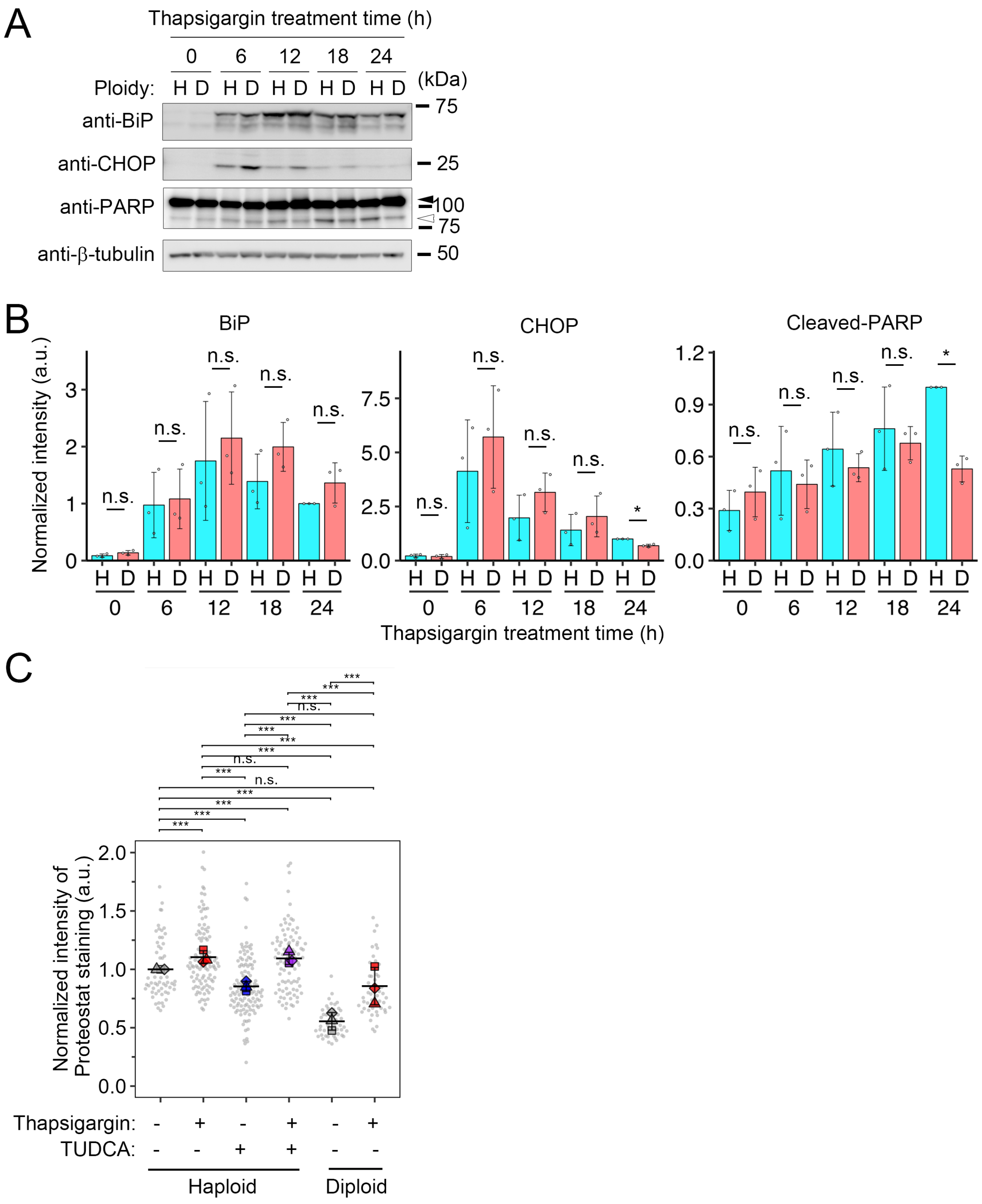

Supplementary figure 5

(A) Immunoblotting of BiP, CHOP, and PARP in haploid or diploid HAP1 cells treated with 10 nM thapsigargin for the indicated duration. The closed or open arrowhead indicates uncleaved or cleaved PARP, respectively. β-tubulin was detected as a loading control. Representative results from 3 independent experiments. **(B)** Quantification of the relative intensity of BiP, CHOP, or cleaved PARP. Protein loading differences were corrected based on β-tubulin signals. Mean ± S.D. of 3 independent experiments. Asterisks indicate statistically significant differences between ploidies (n.s.: not significant, $^{*}p < 0.05$, the Brunner-Munzel test). **(C)** Quantification of cytoplasmic Proteostat signal in haploid or diploid cells treated with or without thapsgarging or TUDCA for 24 h. Mean ± S.D. of 3 independent experiments (mean values within independent experiments are plotted as squares, triangles, or diamonds). Asterisks indicate a statistically significant difference among conditions (n.s.: not significant, $^{***}p < 0.001$, the Steel-Dwass test). At least 48 cells were analyzed for each condition. Single-cell values are also plotted as small grey circles.

Supplementary figure 6

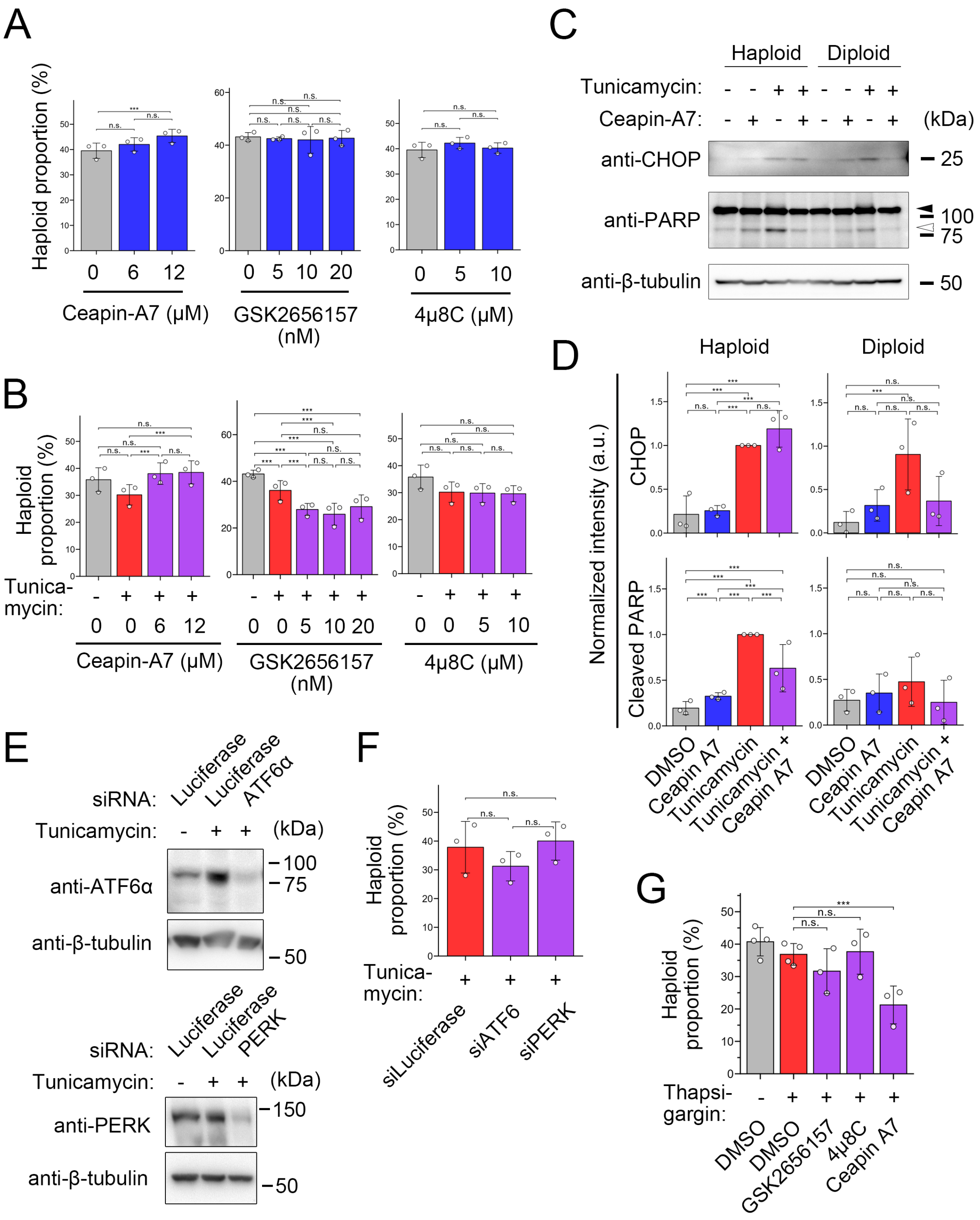

Supplementary figure 6

(A, B, F, G) The proportion of haploid cells in the haploid-diploid co-culture treated with different UPR inhibitors (A), with UPR inhibitors and 50 nM tunicamycin (B), with different siRNAs and 50 nM tunicamycin (F), or with UPR inhibitors and 5 nM thapsigargin (G). Mean ± S.D. of ≥3 independent experiments for each condition. Asterisks indicate statistically significant differences among conditions (n.s.: not significant, $^{***}p < 0.001$, the Steel-Dwass test for A, B, and F, and the Steel test for G). For comparison, the same control data are shown for Ceapin-A7- and 4μ8C-treated samples (A and B). **(C)** Immunoblotting of CHOP and PARP in haploid or diploid HAP1 cells treated with 50 nM tunicamycin with or without 12 μM Ceapin-A7 for 24 h. The closed or open arrowhead indicates uncleaved or cleaved PARP, respectively. β-tubulin was detected as a loading control. Representative results from 3 independent experiments. **(D)** Quantification of the relative intensity of CHOP or cleaved PARP in C. Protein loading differences were corrected based on β-tubulin signals. Mean ± S.D. of 3 independent experiments. Asterisks indicate statistically significant differences among samples (n.s.: not significant, $^{***}p < 0.001$, the Steel-Dwass test). **(E)** Immunoblotting of ATF6α or PERK in RNAi-treated haploid-diploid HAP1 co-culture treated with or without 50 nM tunicamycin used for flow cytometric analysis in F. β-tubulin was detected as a loading control. Representative results from 3 independent experiments.

Table S1: A list of antibodies used in this study

Host and antigen	Identifier and distributor	Dilution	Application
Rabbit monoclonal anti-ATF4	D48B, Cell signaling Technology	1:1000<br>1:500	IB<br>IF
Rabbit monoclonal anti-ATF6	D4Z8V, Cell Signaling Technology	1: 5000	IB
Rabbit monoclonal anti-BiP	C50B12, Cell Signaling Technology	1:1000	IB
Mouse monoclonal anti-CHOP	L63F7, Cell Signaling Technology	1:1000<br>1:500	IB<br>IF
Rabbit polyclonal anti-Grp94	#2104, Cell Signaling Technology	1:1000	IB
Rabbit polyclonal anti-PARP	#9542, Cell Signaling Technology	1:1000<br>1:5000	IB<br>IB (Fig.S6)
Rabbit monoclonal anti-PERK	#3192, Cell Signaling Technology	1:1000	IB
Rabbit monoclonal anti-IRE1	#3294, Cell Signaling Technology	1:1000	IB
Mouse monoclonal anti-PCNA	Sc-56 (PC10), Santa Cruz Biotechnology	1:1000<br>1:10000	IB (Fig. S2A)<br>IB (Fig. S2C)
Rabbit polyclonal anti-cyclin E	Sc-481 (M-20), Santa Cruz Biotechnology	1:100	IB
Rabbit polyclonal anti-cyclin A	Sc-751 (H-432), Santa Cruz Biotechnology	1:100<br>1:1000	IB (Fig. S2A)<br>IB (Fig. S2C)
Mouse monoclonal anti-β-tubulin	10G10, Wako	1:1000<br>1:10000	IB<br>IB (Fig. S6)
Goat peroxidase affinipure anti-rabbit IgG (H+L)	111-035-003, Jackson Immuno Research Laboratory Inc.	1:1000<br>1:5000	IB<br>IB (Fig. S6, PARP)
Goat peroxidase affinipure anti-mouse IgG (H+L)	115-035-003, Jackson Immuno Research Laboratory Inc.	1:1000<br>1:5000<br>1:10000	IB<br>IB (Fig. S6, CHOP)<br>IB (Fig. S6, β-tubulin)
